# User satisfaction with family-based inpatient treatment for adolescent anorexia nervosa: retrospective views of patients and parents

**DOI:** 10.1186/s40337-019-0242-6

**Published:** 2019-05-02

**Authors:** Inger Halvorsen, Øyvind Rø

**Affiliations:** 1 Regional Department for Eating Disorders, Division of Mental Health and Addiction, Oslo University Hospital - Ullevål Hospital, P.O. Box 4956, Nydalen, N-0424 Oslo, Norway; 2 Division of Mental Health and Addiction, Institute of Clinical Medicine, University of Oslo, Oslo, Norway

**Keywords:** Patient satisfaction, Parent satisfaction, Family-based treatment, Inpatient treatment, Anorexia nervosa

## Abstract

**Background:**

Research is scarce on patient and parent satisfaction with family-based treatment for adolescent anorexia nervosa (AN), especially family-based treatment adapted to inpatient settings. The purpose of this study was to describe and compare patient and parent satisfaction with an inpatient family-based treatment program for adolescent AN, and to investigate whether the level of satisfaction with treatment was associated with eating disorder outcome.

**Methods:**

Former patients and their parents were contacted approximately five years (4.5 ± 1.7, range: 1.3–7.0) after discharge from family-based inpatient treatment. Ninety-four participants (patients: *n* = 34, mothers: *n* = 40, fathers: *n* = 20) from 46/58 (79.3%) families took part in the study. Former patients and both parents completed treatment satisfaction questionnaires. Outcome at follow-up was assessed by the Eating Disorder Examination Questionnaire and body mass index (kg/m^2^).

**Results:**

Overall, mothers and fathers reported a high level of satisfaction with treatment, while the former patients’ satisfaction was moderate. There were no significant differences between treatment satisfaction scores for mothers and fathers. However, the former patients’ treatment satisfaction scores were significantly lower than the parents’ scores on several of the items. Correlations between eating disorder outcome parameters and treatment satisfaction were small, except for fathers’ satisfaction with treatment and weight outcome at follow-up.

**Discussion:**

Family-based treatment adapted to inpatient settings is a novel treatment approach for adolescents with AN that require hospitalization. Inclusion and empowerment of parents are considered crucial in outpatient family-based treatment, but may be just as important in inpatient programs. Mothers and fathers alike reported high levels of satisfaction with treatment, which may constitute an important factor in the success of family-based treatment.

**Conclusion:**

Family-based inpatient treatment for adolescents with severe AN who have failed to respond to outpatient treatment seemed to be highly valued by parents and viewed by adolescents as acceptable. Parental satisfaction with their child’s treatment is likely to be an important factor for treatment implementation and adherence both in outpatient and inpatient settings.

## Plain English summary

Knowledge of patients’ and parents’ satisfaction with treatment is important to improve treatment for families affected by serious illnesses such as adolescent anorexia nervosa. The most effective treatment for adolescents with anorexia nervosa is family-based, with a high level of parental involvement. Thus, the parents’ experiences with treatment are particularly important in family-based treatment. However, knowledge of patients’ and parents’ satisfaction with family-based treatment for anorexia nervosa is scarce, particularly in cases when the patient requires hospitalization.

In this study, we investigated the experiences of former patients and their parents following discharge from a family-based, specialized treatment program. Parents physically accompany their child during hospitalization and participate intensively in the treatment. Patients, mothers, and fathers completed treatment satisfaction questionnaires approximately 5 years following discharge. Upon reflection, mothers and fathers expressed high levels of satisfaction with the treatment received. However, as is reflected in other studies on adolescent anorexia nervosa, the parents tended to be more satisfied with treatment than former patients themselves.

## Introduction

Assessment of patient experiences is important for the evaluation and improvement of health services [1–3]. Satisfaction with health services is likely to influence the patient’s engagement in treatment and collaboration with the staff, as well as treatment adherence. Ambivalence to treatment is a major challenge in anorexia nervosa (AN). Patients often report negative treatment experiences [4–6] and have a high dropout rate [7, 8]. However, dropout from family therapy for AN has been found to be much lower than from individual therapy [8]. Because the parents have a crucial role in ensuring that their children seek and adhere to treatment, the parents’ views and satisfaction with treatment may be more important for a successful delivery and implementation than the patients’ own satisfaction [9, 10].

Family-based treatment (FBT) [11, 12] is the most well-documented treatment for adolescent AN [13, 14]. Originally, FBT was developed as an outpatient intervention. However, many adolescents with AN need to be hospitalized due to severe physical complications, failure to respond to outpatient FBT, or lack of access to adequate outpatient treatment. When outpatient FBT is insufficient, FBT adapted to more intensive levels of care might be beneficial for adolescents with AN [15–19].

An important challenge when adapting outpatient FBT to inpatient settings is to combine FBT’s strong focus on empowering the parents with the highly structured framework in inpatient programs that may be deemed necessary to manage the patient’s medical condition [15]. During family-based hospital admissions, the parents are included in the daily care and intensive treatment and are able, therefore, to get more extensive coaching and support than what is possible in outpatient settings [16, 17]. As in outpatient FBT, the staff will support the parents’ authority, but, typically, the inpatient staff will assume more responsibility for monitoring the patient’s medical condition and for the framework in the treatment program. Adolescents with AN who require hospitalization will be expected to have a more severe prognosis than those in outpatient care, but family-based inpatient treatment may be associated with better outcomes than individual admissions [16, 18]. For instance, one study that compared outcome after an inpatient FBT intervention for medical stabilization in youths with AN with “treatment as usual” (TAU) hospitalization, found that those who received the FBT intervention were 2.84 times more likely than the TAU-group to achieve at least 95% of treatment goal weight at 6 months post-discharge [16]. Also, parental self-efficacy increased significantly in caregivers who participated in the FBT intervention [16]. The close collaboration with parents in family-based inpatient treatment may be important to reduce dropout.

Despite the parents’ key role in the treatment, only a few studies have investigated parental satisfaction with their child’s ED treatment [20–24], or with interventions aimed at parents [24, 25]. None of these studies has investigated parental satisfaction with family-based inpatient treatment. A study from the United Kingdom, for example, investigated patients’ and parents’ satisfaction with three types of services for adolescent AN [20]. Results showed that parental satisfaction was highest for specialized outpatient services, intermediate for specialized inpatient services, and lowest for unspecialized child and adolescent mental health services [20]. Research on family-based treatment for EDs have generally found high levels of parental satisfaction with treatment for adolescent AN, and interestingly, parents tend to be more satisfied than the patients themselves [20, 21]. Supporting parents’ efforts to help their child is often underscored as an important aspect of care related to greater satisfaction [24, 25].

A core aspect of FBT involves the inclusion of both parents in treatment, provision of support, and close collaboration in helping their child to overcome the ED [11, 26]. In addition to a high level of maternal involvement, the extent of fathers’ participation in FBT has been found to predict better outcome at the end of treatment [27]. Also, the agreement between mothers and fathers in their perceptions of treatment may influence their collaboration in coping with the ED. To our knowledge, only one previous study has compared mothers’ and fathers’ perceptions of their child’s AN treatment [21]. In that study, both fathers and mothers reported an overall positive perception of their child’s treatment, with a high level of parental agreement.

Knowledge is scarce and inconsistent as to whether patient and/or parent satisfaction with treatment for adolescent AN is associated with better ED outcome. In a study by Paulson-Karlsson et al. [23], there was no statistically significant difference in treatment satisfaction between recovered and not-recovered patients. However, the parents of recovered patients reported significantly higher treatment satisfaction than parents of non-recovered patients. A study by Halvorsen & Heyerdahl [21] found that patient and parental treatment satisfaction was not significantly associated with whether the patient still suffered from an ED at follow-up assessment.

The primary aim of the current study was to describe and compare how former patients, mothers, and fathers perceived and experienced family-based inpatient treatment for adolescent AN following discharge. Specifically, areas covered included the effectiveness or perceived benefit of treatment, perceptions of the therapists, and fulfillment of treatment goals at follow-up. Furthermore, a secondary aim was to investigate whether outcome (i.e., weight gain (kg) from admission to follow-up, BMI, and eating disorder psychopathology at follow-up) were related to patient and parent treatment satisfaction.

## Methods

This was a sub-study of a larger follow-up of families who received family-based inpatient treatment for adolescent AN at the Regional Department for Eating Disorders at Oslo University Hospital during the period 2008–2014 [17]. Patients were referred from specialized mental health services and all had received previous inpatient and outpatient treatment at their local child- and adolescent mental health services. This had included family-based interventions, typically focusing on parental support to eat, but usually not manualized FBT. At the child and adolescent inpatient unit (for patients up to 18 years old), the standard treatment program is family-based treatment [11, 12] which has been adapted for delivery at an inpatient setting. This approach was selected based upon evidence supporting the effectiveness of FBT for adolescent AN in outpatient settings [13]. The unit has beds for a maximum of five patients to be admitted with their parents (and siblings when applicable).

Children under 18 years of age in Norway have the right to be accompanied by a parent during hospital stays because proximity to primary caregivers is considered important for the health and well-being of sick children and adolescents [28]. Parents/guardians in Norway receive financial benefits to stay with children under 18 years of age during hospitalization, which is usually equivalent to full salary. All families who participated in the present study had at least one parent who agreed to stay at the hospital with their child during the admission.

### Participants and procedure

All former patients (*n* = 58) and parents (*n* = 104) who received family-based inpatient treatment at the unit during the period May 2008–June 2014 were contacted via a letter of invitation sent to each family member asking them to participate in the study. Non-responders were reminded by mail and/or telephone. In the case of re-admissions to the same unit, the focus of the follow-up was based upon the initial family admission. Only one family discontinued treatment against recommendations from the medical treatment team. At admission, all patients had a diagnosis of AN, with the exception of one patient who was admitted with a bulimia nervosa diagnosis despite a past history of AN. Mean age at admission was 15.8 ± 1.8 years, mean BMI percentile (for age and sex) 4.9 ± 15.4, and mean duration of illness 2.8 ± 1.8 years. Mean duration of the family admission was 20.3 ± 12.9 weeks, mean weight gain during the admission 7.1 ± 4.5 kg and mean discharge BMI percentile 20.9 ± 20.6.

The sample included 34 former patients, 40 mothers, and 20 fathers from 46 (79%) of the 58 eligible families. There were 31 mother/patient pairs, 18 mother/father pairs and 12 father/patient pairs available for the analyses. The number of participants from each family ranged from one to three. When the participants had provided written consent, they completed the follow-up assessment packet at home and brought it along to an interview conducted by an experienced clinician. No statistically significant differences existed between the 46 participating and the 12 non-participating families for the following background variables: duration of ED before admission, age at admission, admission BMI percentile, weight gain during the admission, discharge BMI percentile, age at the time of the follow-up study, and time between discharge to follow-up.

### Treatment

Treatment principles from the Maudsley FBT [11, 12, 29] were adapted and modified for delivery on an inpatient setting. As in FBT-adapted day treatment programs [18] and traditional day treatment, the inpatient program was intensified compared to outpatient care to meet the needs of patients with severe medical conditions and/or unsatisfactory response to outpatient treatment. While day treatment, or partial hospitalization, programs may be delivered from a few hours to several days a week, and parents usually attend only part of that time [18], inpatient family-based treatment entails that one or both parents would stay at the hospital during the entire period to take part in the intensive daily care and treatment of their child [16, 17].

If the parents lived together (*N* = 31, 66% of the families), both parents were encouraged to stay at the unit to facilitate parental communication and to develop cooperative skills in managing the illness. Typically, both of the parents stayed at the unit during the first phase of the treatment, and thereafter alternated between staying at the hospital versus at home with siblings. If the parents did not live together (*N* = 15, 33%), one of the parents stayed at the hospital at a time, while both participated in treatment meetings, parental counseling, and family sessions. In nine (20%) of the families, only one parent was able to participate in treatment, typically due to health problems affecting the other parent or in the case of sole custody.

Most of the inpatient admission corresponds to the first phase of the Maudsley approach, where the main focus is on helping the parents take charge of their child’s weight restoration. In our inpatient setting, the staff assumed more responsibility than in outpatient FBT to provide a structured regime for weight gain, i.e., by providing meal plans, prescribing supplemental nutritional drinks, or in seldom cases tube-feeding, if the patient did not finish her/his meal, and recommending how much activity and rest the child needed. The more extensive support was provided to help ensure adequate weight gain, as previous inpatient and outpatient treatment had not led to remission. Treatment was administered in close collaboration with the parents, and with a clear goal to support the parents’ role in caring for their child both emotionally and with overcoming the ED.

The family ate all meals together in a designated family room or at a family table in the dining room. The parents were responsible for helping their child to eat sufficiently, while the staff’s role was to support the parents to accomplish this task rather than taking over the responsibility for the child’s eating. If the parents needed help during the meal, one of the staff members would encourage them to trust in themselves, to not give up and to support each other, but also offer consultation and specific advice, for example, on how to articulate and repeat the message that the food must be eaten. Milieu therapy staff aimed to support the whole family in coping with the ED, reducing conflicts, promoting cooperation and facilitating positive relationships between family members, and empowering parents to support their sick child. During the last phase of hospitalization, when most patients had regained normal body weight, the focus gradually shifted toward encouraging the adolescent to assume greater responsibility for eating and exercise with continued parental supervision, that is, to help themselves to food from the buffet or negotiate how to resume physical activities. This transition corresponds to the second phase in Maudsley FBT.

Physical health was closely monitored, and a weekly weight gain of approximately one kg was recommended. The individual treatment plan was reviewed and adjusted at weekly treatment meetings. Both parents attended treatment meetings, and occasionally, staff members from the referring local child- and adolescent mental health services also attended. The family spoke with the staff on a daily basis to review progress and attended joint family sessions once or twice a week. The family sessions included core elements from outpatient FBT/Maudsley approach, but was adapted to our inpatient setting and to the families’ situation, which was characterized by long duration and severity of AN as well as previously unsuccessful treatment. Parents also attended weekly group sessions for parents, where they could share experiences and support one another, and meetings alone with staff members. Supportive individual sessions were offered weekly to patients if requested. During some periods, group sessions for patients and a sibling group was also offered. Finally, weekend leaves from the hospital were integrated into treatment to encourage patients and parents to practice and transfer skills to the home environment, with longer leaves granted toward the end of hospitalization. At discharge, the patients were referred to further outpatient treatment at their local mental health services. Thirty (88%) had received outpatient treatment, and 13 (38%) had been rehospitalized, during the follow-up period.

**Data from the treatment phase** were collected from patient records.

### Assessment at follow-up

ED diagnoses and body weight were assessed by an experienced clinician at the patient interview [17].

#### Body weight and height

Body mass index (BMI, kg/m^2^) was calculated from either measured (*n* = 16) or self-reported (*n* = 18) weight and height. Self-reported weight and height were used if the participant did not want to be weighed and when the interview took place at home or by telephone. Mean BMI based on measured weight/height (19.8 ± 3.3) did not differ from that based on self-report (19.9 ± 2.9, n.s.) in the group with only self-reported BMI.

#### Assessment of ED symptoms

The Eating Disorder Examination Questionnaire 6.0 (EDE-Q) [30] is a widely-used 28-item self-report measure of ED psychopathology based on the EDE interview. The EDE-Q focuses on core ED symptoms and behaviors, including binge eating and purging, during the past 28 days. It is comprised of four subscales: dietary restraint, eating concern, weight concern, and shape concern. The mean of the subscale scores comprises a global EDE-Q score. An EDE-Q global score < 2.5 has been demonstrated as the clinical threshold to optimally distinguish non-cases from cases in a sample of Norwegian women [31]. The Norwegian version of the EDE-Q has shown adequate psychometric properties in community and clinical samples, with good convergent validity between the EDE and EDE-Q, and good internal consistency (Cronbach’s Alpha coefficients 0.76–0.90) and inter-rater reliability (0.70–0.97) for the four subscales [32].

#### Assessment of treatment satisfaction

The following questionnaires were administered to former patients, mothers and fathers:

- General satisfaction with treatment: The Norwegian Generic Short Patient Experiences Questionnaire (GS-PEQ) [1, 33] covers important aspects of user experiences across a range of specialist health services and is widely used in Norwegian health services. The questionnaire consists of 14 items with five response categories ranging from “not at all satisfied” to “very satisfied” (see Table 1). The GS-PEQ has shown good evidence for validity and reliability, good internal consistency (Cronbach’s Alpha coeffiecient > 0.70) and good test-retest reliability (intraclass correlations between 0.62–0.85 for the different items) [34].
- Perceived benefit of treatment: One item from the GS-PEQ on overall benefit of treatment was administered (Table 1, a). The test-retest reliability of this single item was found to be 0.73 [33]. Additionally, two items were designed by the authors to assess overall usefulness of treatment for other family members (Table 1, b and c) as well as items on the perceived utility of 19 different treatment aspects, with five response categories ranging from “no benefit” to “very large benefit”.
- Perception of therapists (POT): A treatment satisfaction questionnaire from the Swedish National Quality Register on EDs [21, 35] was translated into Norwegian by the first author. The instrument consists of 11 items on the perceptions of the therapists, which in this context was defined as all staff members who participated in the treatment, plus two items for parents that were added for this study (Table 2). The instrument has six response categories ranging from “never” to “always”.
- Treatment goals: Another questionnaire from the same Swedish National Quality Register [21, 35] was used, and was translated into Norwegian by the first author. The instrument includes ten goals for ED treatment that were rated according to the degree to which each goal was perceived as fulfilled (four response categories ranging from “not at all” to “yes, completely”) (Table 3). The original instrument also had questions about each goal’s relevance and importance, but these were not used in the present study.

**Table 1:** The family members’ general experiences with, and benefit of, family inpatient treatment

	Mean score ± SD			Patients /moth.^b^		Patients./fath.^c^		Moth./fath.^d^	
General experiences with the treatment^a^	Patients *N* = 34	Mothers *N* = 40	Fathers *N* = 20	*P*-value^e^	Effect size^f^	*P*-value^e^	Effect size^f^	*P*-value^e^	Effect size^f^
Mean general experiences with treatment score (item 1–12)	3.2 ± 1.0	3.7 ± 0.7	3.9 ± 0.5	0.001	0.6	0.029	0.9	0.457	0.3
1. Did you have confidence in the clinicians’ professional competence?	3.3 ± 1.4	4.1 ± 0.8	4.2 ± 0.5	0.006	0.7	0.117	0.9	0.718	0.1
2. Did the clinicians talk to you in a way that was easy to understand?	3.8 ± 1.0	4.4 ± 0.7	4.1 ± 0.6	0.001	0.7	0.236	0.4	0.579	0.5
3. Did you get sufficient information about the illness/diagnosis?	3.6 ± 1.1	3.9 ± 0.9	3.9 ± 0.8	0.008	0.3	0.242	0.3	0.651	0.0
4. Did you perceive the treatment as suited to your situation?	2.9 ± 1.4	3.5 ± 1.2	3.9 ± 0.9	0.009	0.5	0.034	0.8	0.311	0.4
5. Were you involved in decisions regarding your/your child’s treatment?	2.9 ± 1.2	3.7 ± 1.1	3.5 ± 0.9	0.038	0.7	0.191	0.7	0.529	0.3
6. Did you perceive the institution’s work as well organized?	3.4 ± 1.3	3.9 ± 1.0	3.9 ± 0.7	0.055	0.4	0.223	0.5	0.632	0.0
7. Was the staff aware of siblings’ situation?^g^	2.4 ± 1.2	3.2 ± 1.0	3.7 ± 0.8	0.003	0.7	0.120	1.3	0.510	0.6
8. Did the staff collaborate well with the parents?	3.4 ± 1.4	4.1 ± 0.8	4.1 ± 0.6	0.021	0.6	0.296	0.6	0.632	0.0
9. Did the staff collaborate well among themselves?	3.6 ± 1.2	4.0 ± 0.9	4.0 ± 0.5	0.131	0.4	0.312	0.4	0.458	0.0
10. Did the staff cooperate well with other public services?	3.2 ± 1.1	3.4 ± 1.0	3.8 ± 0.7	0.282	0.2	0.082	0.7	0.111	0.5
11. Did you perceive that the institution prepared you for the time after discharge?	2.6 ± 1.4	3.0 ± 1.1	3.3 ± 0.9	0.142	0.3	0.468	0.6	0.718	0.3
12. Overall, was the help and treatment you/ your child received satisfactory?	2.9 ± 1.3	3.7 ± 1.0	4.1 ± 0.9	< 0.001	0.7	0.001	1.3	0.333	0.6
Mean score on overall benefit of the treatment for family members (a-c)^h^	3.1 ± 1.0	3.3 ± 1.1	3.5 ± 1.1	0.019	0.2	0.046	0.4	0.913	0.2
a) Overall benefit for the patient	3.0 ± 1.1	3.6 ± 1.1	3.6 ± 1.2	< 0.001	0.5	0.016	0.5	0.333	0.0
b) Overall benefit for the parents	3.5 ± 1.1	3.6 ± 1.1	3.7 ± 1.0	0.147	0.1	0.795	0.2	0.331	0.1
c) Overall benefit for siblings^g^	2.7 ± 1.3	2.4 ± 1.3	3.0 ± 1.2	0.843	0.2	0.457	0.2	0.305	0.5
Mean score on benefit of 19 different treatment elements^g^	3.0 ± 0.9	3.4 ± 0.9	3.4 ± 0.8	0.042	0.4	0.218	0.5	0.128	0.0

^a)^Reply options: not at all, to a small extent, to a moderate extent, to a large extent, to a very large extent. Possible range:1–5, more satisfied the higher score, ^b)^ 31 pairs, ^c)^ 12 pairs, d^)^18 pairs, ^e)^paired samples t-tests, ^f)^Cohen’s d (small: 0.2. medium: 0.5, large: 0.8), ^g)^applicable for *n* = 25 patients, *n* = 35 mothers and *n* = 19 fathers, ^h)^reply options: no benefit, little benefit, some benefit, large benefit, very large benefit. Possible range:1–5, more satisfied the higher score

**Table 2:** Perception of the contact with the therapists/staff members who participated in the treatment (POT) by patients and their parents

	Mean score ± SD			Patient/mother^c^		Patient/father^d^		Mother/father^e^	
	Patient *N* = 34	Mother *N* = 40	Father *N* = 20	*P*-value^f^	Effect size^g^	*P*-value^f^	Effect size^g^	*P*-value^f^	Effect size^g^
Mean POT^a^ score (item 1–11)	3.6 ± 1.2	4.6 ± 0.9	4.8 ± 0.7	< 0.001	0.9	< 0.001	1.2	0.227	0.2
Do you think that the therapist(s)^b^:									
1. Understood your problems?	3.3 ± 1.4	4.5 ± 1.0	4.5 ± 0.6	< 0.001	1.0	0.007	1.1	0.751	0.0
2. Received you well?	4.1 ± 1.4	4.8 ± 0.9	4.9 ± 0.8	0.002	0.6	0.001	0.7	0.261	0.1
3. Respected you as a person?	4.2 ± 1.5	4.9 ± 1.1	5.2 ± 0.8	0.002	0.5	0.003	0.8	0.264	0.3
4. Let you to talk about what was important to you?	4.1 ± 1.6	4.7 ± 1.1	5.0 ± 0.6	0.018	0.4	0.013	0.7	0.164	0.3
5. Listened to you?	4.1 ± 1.5	4.6 ± 1.2	5.0 ± 0.7	0.103	0.4	0.002	0.8	0.188	0.4
6. Were able to help you/your daughter/son?	3.6 ± 1.6	4.1 ± 1.3	4.2 ± 1.3	0.022	0.3	0.017	0.4	0.751	0.1
7. Gave your parents/you enough help to support you/your daughter/son in overcoming the ED?	3.0 ± 1.4	4.6 ± 1.2	4.7 ± 0.9	< 0.001	1.2	0.001	1.4	0.387	0.1
8. Agreed with you on the treatment goal?	2.9 ± 1.5	4.8 ± 1.0	4.7 ± 1.0	< 0.001	1.5	< 0.001	1.4	0.721	0.1
9. Agreed with you on how the treatment should be?	2.9 ± 1.4	4.4 ± 1.0	4.5 ± 0.9	< 0.001	1.2	< 0.001	1.4	0.586	0.1
10. Had sufficient knowledge of ED and knew what they were doing?	3.8 ± 1.6	4.9 ± 1.1	4.9 ± 1.0	< 0.001	0.8	0.002	0.8	1	0.0
11. Valued your own efforts in overcoming the ED?	3.6 ± 1.7	4.7 ± 1.2	4.8 ± 1.0	0.001	0.7	0.001	0.9	0.301	0.1
For parents only:									
12. Were able to help you as a mother/father?		4.1 ± 1.2	4.3 ± 0.8					0.818	0.2
13. Gave you enough help to support each other as parents?		3.9 ± 1.4	4.5 ± 1.1					0.302	0.5

^a)^ Perception of therapists, ^b)^reply options: never, seldom, sometimes, often, very often, always. Possible range:1–6, more satisfied the higher score, ^c)^ 31 pairs, ^d)^ 12 pairs: *n* = 24, e^)^ 18 pairs,, ^f)^paired samples t-tests, ^g)^Cohen’s d (small: 0.2, medium: 0.5, large: 0.8)

**Table 3:** Fulfillment of treatment goals

	Mean score ± SD			Patients /moth.^b^		Patients./fath.^c^		Moth./fath.^d^	
	Patients *N* = 34	Mothers *N* = 40	Fathers *N* = 20	*P*-value^e^	Effect size^f^	*P*-value^e^	Effect size^f^	*P*-value^e^	Effect size^f^
Mean goal fulfillment score (mean of the 10 potential goals)	2.1 ± 0.7	2.4 ± 0.6	2.6 ± 0.6	0.006	0.5	0.019	0.8	0.527	0.3
Potential treatment goals: Was the goal fulfilled?^a^									
1. To learn more about eating disorders	2.4 ± 0.9	2.8 ± 0.7	2.9 ± 0.6	0.094	0.5	0.045	0.7	0.775	0.2
2. To get help talking about painful experiences	2.3 ± 1.1	2.4 ± 0.7	2.6 ± 0.7	1.000	0.1	0.799	0.3	0.336	0.3
3. Learn to eat normally	2.3 ± 1.0	2.4 ± 0.9	2.4 ± 0.8	0.326	0.1	0.760	0.1	0.720	0.0
4. Learn to cope with unreasonable notions about food and body size	1.8 ± 0.7	2.3 ± 0.8	2.4 ± 0.9	0.008	0.7	0.081	0.7	0.190	0.1
5. Become more satisfied with myself and my body	1.5 ± 0.7	2.1 ± 0.8	2.3 ± 0.9	< 0.001	0.8	0.019	1.0	0.426	0.2
6. To get help to stand up for what I feel / she or he feels	2.0 ± 1.0	2.4 ± 0.7	2.6 ± 0.6	0.036	0.5	0.104	0.7	0.336	0.3
7. Reduce guilt and self-blame	1.7 ± 0.8	2.2 ± 0.7	2.3 ± 0.9	0.002	0.7	0.021	0.7	0.793	0.1
8. To get support in crisis situations	2.6 ± 1.1	2.8 ± 0.9	2.8 ± 1.0	0.231	0.2	0.321	0.2	0.613	001
9. Get help to cope with difficult feelings such as sadness, anxiety and anger	2.2 ± 1.0	2.4 ± 0.8	2.4 ± 0.7	0.183	0.2	0.563	0.2	1.000	0.0
10. To reduce conflicts in the family related to difficulties with eating	2.1 ± 0.9	2.5 ± 0.8	2.6 ± 0.8	0.004	0.5	0.049	0.6	0.673	0.1

^a)^ Range: 1–4, the higher score the more fulfilled^.^ Reply options: 1 = not at all, 2 = only to some extent, 3 = yes, mostly, 4 = yes, absolutely, ^b)^ 31 pairs, ^c)^ 12 pairs, ^d)^18 pairs, ^e)^paired samples t-tests, ^f)^Cohen’s d (small: 0.2, medium: 0.5, large: 0.8)

### Statistical analyses

Data were fairly normally distributed and parametric analyses were used. Independent t-tests were used to investigate differences in background variables between participants and non-participants. Differences between former patients, mothers, and fathers on the treatment satisfaction measures were investigated using paired samples t-tests. Bivariate associations between continuous variables were investigated with Pearson’s correlations. All analyses were two-tailed. When multiple analyses were used, the alpha level was set at *p* < .01.

The study was approved by the Regional Medical Ethics Committee.

## Results

The average time between discharge and follow-up was 4.5 ± 1.7 years (range 1.3–7.0). Mean age of the former patients was 20.4 ± 2.7 years. Twenty-one (62%) of the participants were weight recovered (i.e., BMI > 18.5) and 13 (38%) fully recovered (i.e., normal eating attitudes and behavior defined as an EDE-Q global score < 2.5, no binge eating/purging during the past three months, and BMI > 18.5).

### General experience with treatment

Overall, parents reported a high level of satisfaction with all central aspects of treatment, including perceived competence of the therapists, communication and collaboration (Table 1). There were no significant differences between mothers and fathers in their general experience of treatment. Former patients had significantly lower scores than mothers and fathers on the items, “Did you perceive the treatment as well-suited to your situation?” and “Overall, was the help and treatment you/your child received satisfactory?” In addition, former patients had significantly lower mean scores on the 12 general experience items. Effect sizes indicated moderate to large differences in scores between parents and former patients (Table 1). The mean score for former patients’ “general experience with treatment” was 3.2 (±1.0), reflecting a medium level of satisfaction. All family members had the lowest scores on the item: “Did you perceive that the institution prepared you for the time after discharge?”

Five percent of mothers and fathers replied that they thought their child had “in any way received the wrong treatment”, in contrast to 15% of the former patients.

### Benefit of treatment

The former patients rated their own overall benefit from treatment significantly lower than the parental ratings of the overall benefit for their child (medium effect sizes, Table 1). However, former patients and parents had similar views on the overall benefit of treatment for parents. Parents and former patients also had similar, but lower, ratings on the overall benefit for siblings (Table 1). Table 1 illustrates the mean scores for the perceived benefit of 19 different treatment elements.

### Perception of the contact with the therapists/staff members

Mothers and fathers alike reported a similarly high level of satisfaction with therapist contact (Table 2). The former patients, however, perceived the contact with therapists more negatively than mothers or fathers, with medium-to-large effect sizes. The former patients gave the highest ratings to being well-received, respected, allowed to talk about what was important to her/him, and being listened to (> 4, corresponding to “often”). The lowest scores (< 3, corresponding to “sometimes”) related to agreement on treatment goals, agreement on treatment structure/content, and the help their parents had received to support their recovery from an ED.

### Fulfillment of treatment goals

Ten possible goals for ED treatment were rated according to the extent to which the goal was perceived as fulfilled (Table 3). Former patients had a significantly lower overall mean goal fulfillment score than mothers and fathers (medium-large effect sizes) as well as poorer ratings for several of the specific goals. Receiving “help in crisis situations” and “learning more about EDs” were perceived by all participants (former patients, mothers, and fathers) as the most fulfilled goals. Increased satisfaction with body/self, reduction of guilt/shame, and learning to cope with unrealistic beliefs regarding food and body weight/shape were perceived as the least fulfilled goals by all groups.

### Correlations between treatment satisfaction measures and outcome variables

As shown in Table 4, correlations were performed to investigate associations between the various aspects of treatment satisfaction and the three ED outcome variables (weight gain from admission to follow-up, BMI and EDE-Q global score at follow-up). Among former patients, all correlations between treatment satisfaction and the ED outcome variables were low (r < 0.17, n.s.). Among mothers, all correlations were low except the correlation between mothers’ average goal fulfillment score and the two outcome variables weight gain from admission to follow-up (r = 0.49, *p* = 0.008) and BMI at follow-up (r = 0.56, *p* = 0.002). In contrast, for the fathers, all the treatment satisfaction measures showed large correlations with weight gain from admission to follow-up and BMI at follow-up, but not with the EDE-Q global score (Table 4).

**Table 4:** Correlations between treatment satisfaction variables and ED outcome variables^a^

		ED outcome variables		
		Weight gain from admission to follow-up	BMI At follow-up	EDE-Q global score At follow-up
Weight gain from admission to follow-up		1		
BMI at follow-up		0.82*	1	0.02
EDE-Q global score at follow-up		0.10	0.02	1
Mean general experiences with treatment score (see Table 1)	Patient	0.08	0.11	0.09
	Mother	0.28	0.19	0.10
	Father	0.66	0.68	0.20
Mean score on overall benefit of the treatment for family members (see Table 1)	Patient	0.16	0.17	0.03
	Mother	0.20	0.16	0.16
	Father	0.72*	0.62	−0.03
Mean Perception of therapist (POT) score (see Table 2)	Patient	0.06	0.17	−0.07
	Mother	0.17	0.15	0.07
	Father	0.67	0.72	−0.08
Mean goal fulfillment score (see Table 3)	Patient	−0.05	0.05	−0.07
	Mother	0.49*	0.56*	−0.07
	Father	0.84*	0.85*	0.15

^a)^Pearson’s correlations. Number of pairs: between patient variables: 34, patient/mother: 31, patient/father: 12

*p < 0.001

## Discussion

Despite the crucial role that parents play in treatment engagement and adherence for their child, research is scarce on how parents perceive and experience treatment for adolescent AN. This study investigated patient and parental satisfaction with family-based inpatient treatment for adolescent AN as assessed retrospectively at approximately five years (4.5 ± 1.7 years) following discharge.

Several main conclusions can be derived from our findings. First, mothers and fathers alike reported similar and overall positive experiences with treatment, whereas the former patients were significantly less satisfied than parents, reporting a medium level of overall satisfaction. Second, we found no strong support for the notion that treatment satisfaction was associated with outcome of the ED. Eating disorder symptomology as measured by the EDE-Q demonstrated very low correlations (r < 0.2, n.s.) with the various aspects of treatment satisfaction rated by patients and parents.

Our finding that parents reported a higher level of satisfaction with their child’s AN treatment than the patients themselves is in accordance with three other studies comparing parents’ and patients’ treatment satisfaction [20, 21, 23]. The very low dropout rate from treatment in the present study (1/58 families) may be related to the high level of parental satisfaction. Overall, mothers and fathers reported rather similar perceptions of treatment, which might be a positive factor for collaboration and consistency between the parents in their efforts to help their child. In terms of perceived benefit of treatment for the child, former patients showed lower ratings than parents, however, all family members rated the overall benefit for parents as good (Table 1). FBT emphasizes that the parents, not the patient, must take charge of their child’s weight restoration, and, thus, the parents’ benefit of treatment may be an important factor for outcome. One reason for FBT’s superiority compared to individually-based treatment for adolescent AN may be that the treatment is less affected by the young patient’s own ambivalence and fear of weight gain.

Studies of patient satisfaction with health services tend to find that most patients report a high level of satisfaction [33, 36], which is consistent with the present findings for parents, but not the patients. AN is a highly ego-syntonic disorder for those personally afflicted by the illness, but not for parents, and this may differentially influence patients’ and parents’ perceptions of treatment [37, 38]. Extreme fear of eating and weight gain are core symptoms of AN, and the patients usually experience particularly strong ambivalence to treatment components directly aiming to restore a healthy body weight [38]. Weight restoration is a main overarching goal for hospitalization and, thus, it may be natural that patients, even in retrospect, might experience their ED treatment as difficult and challenging, with lower treatment satisfaction ratings than their parents.

Lower patient scores were observed for items related to agreement with treatment goals and treatment content/structure, with average scores of 2.9 (corresponding to “sometimes”). As several aspects of treatment were non-negotiable, such as the amount of food, amount of rest and physical activity, this finding is perhaps expected. Unfortunately, we do not know whether former patients desired greater influence on their treatment plan, or if they retrospectively felt that parents and therapists needed to manage these aspects of treatment.

Various aspects of the therapeutic relationship, such as being well-received, respected, listened to, and having the freedom to discuss personally important topics received the highest treatment satisfaction ratings from former patients. This finding is consistent with other studies that have found that patients with AN tend to be satisfied with their relationship with therapists [39], but less satisfied with treatment goals relating to restoration of a healthy body weight, which are goals typically viewed as essential by healthcare professionals [38, 40]. Suffering from AN during adolescence is inarguably extremely difficult and distressing, and it is encouraging that the majority of former patients retrospectively perceived their relationship with the therapists positively, despite the lower rating given for other aspects of treatment.

Inpatient treatment has been shown to be effective in achieving weight gain, but less effective in improving psychological symptoms [41, 42]. Consistent with these findings, our sample of former patients, mothers, and fathers reported the lowest goal fulfillment scores for reducing psychological ED symptoms, e.g., body dissatisfaction, and intermediate scores for the item “learning to eat normally”. Outcome studies of FBT for adolescent AN have found that the majority of the patients successfully achieved weight recovery by the end of treatment and/or follow-up, but that a lower proportion achieved normal eating attitudes [13]. For instance, in a randomized treatment study by Lock et al. [43] only 34% of participants who had received FBT, and 20% that had received individual therapy, had achieved both EDE-Q global scores in the normal range and full weight-restoration at one year follow-up. Consistent with other studies [13], a minority (38%) was shown to meet the criteria for full recovery at follow-up in our study despite the rather long follow-up time (4.5 years) [17].

It might have been expected that patients with a favorable ED outcome and their parents would be comparatively more satisfied with treatment. However, in line with two other studies [21, 23], we did not find any strong evidence to support this assertion. Our results suggested no clear picture of the relationship between treatment satisfaction and degree of improvement or outcome at follow-up. Some were satisfied with treatment despite having a poor ED outcome, while others were less satisfied despite having a favorable outcome, perhaps believing they had managed to overcome the illness despite unsatisfactory treatment. It is possible that patients tend to value the therapeutic relationship, while aspects of treatment that directly tackle weight-restoration, despite being necessary to recover from AN, are associated with ambivalence and reluctance.

Parental therapeutic alliance in FBT has been found to be associated with weight gain, which parents have control over in FBT, but less associated with cognitive improvement that parents have less control over [44]. Unlike mothers and former patients, the fathers’ ratings of treatment satisfaction correlated strongly with the patients’ weight outcome. Thus, weight recovery may have contributed to treatment satisfaction among fathers, or, vice versa, that fathers with high levels of treatment satisfaction contributed to effective weight restoration [44]. Further research should investigate whether fathers emphasize the importance of achieving a healthy body weight to a greater extent than mothers or patients. This would be consistent with a study on fathers’ views on parental interventions which found that fathers tended to prefer a focus on measurable outcomes [45]. It is possible that fathers may experience even stronger feelings of helplessness than mothers when their child is affected by AN and that they particularly appreciated the focus on strengthening specific parental skills in helping their underweight child gain weight during the family-based treatment. Fathers may also feel that mental health services are focused on the mother and perhaps consider the mother’s competence and ability to provide care as more important [45]. One study found that the mothers’ attendance in FBT-sessions for adolescent AN was much higher than fathers’, but that a higher rate of attendance by fathers predicted better outcome [27], suggesting the importance of extensive involvement of both mothers and fathers in AN treatment.

The present study provides insight into patient and parental satisfaction with family-based inpatient treatment for adolescent AN, which is a novel treatment approach with comparatively less research attention. Moreover, studies of patient and parental satisfaction for treatment of adolescent AN is scarce, despite the importance to improve available health services. The inclusion of both parents is considered essential in FBT, but there is a lack of studies on how mothers and fathers experience treatment. The present study included several measures of treatment satisfaction, and compared satisfaction between former patients, mothers, and fathers.

Nevertheless, this study had several limitations that should be noted. The number of participants was small, rendering statistical power low. The number of participants from the same families varied, which limited the numbers of available pairs to investigate mean differences or correlations. In particular, the available patient-father pairs were low (*n* = 12 pairs), which renders uncertainty about the representativeness of the results. Thus, these findings should be considered preliminary and require replication. Treatment satisfaction was assessed at follow-up, following an average of 4.5 years after discharge, which is likely to have affected how the participants recalled the treatment. The majority of the patients had received further treatment during the follow-up period, which also might have influenced their retrospective perceptions of the family inpatient program. “General satisfaction with treatment” was assessed with an instrument with well-established psychometric properties, although data on reliability or validity were unavailable for two of the treatment satisfaction questionnaires (“Perception of the contact with the therapists” and “Treatment goals”). Furthermore, the study was conducted at the institution that had delivered the treatment, which might have influenced the family members’ responses.

Family admissions are demanding both for health service providers and parents, and the generalizability of findings related to this treatment approach may be limited by a lack of social welfare systems to compensate for parents’ loss of income during their child’s illness. However, individual admissions also demand extensive resources from families and the health services. If individual admissions are shown to be less effective than family admissions, resulting in prolonged parental distress and burden of care [46, 47], this could adversely affect parents’ health and work ability, surpassing the financial toll incurred during a family admission. When an adolescent with AN fails to remit despite receiving outpatient FBT, the parents need extensive support to regain trust in their own ability to help their child [48]. Individual admissions might be less beneficial for the parent-child relationship, mother-father collaboration, and their ability to cope with the ED, both during and after the hospital stay. Thus, greater knowledge is warranted about inpatient approaches that include parents in the treatment of adolescents with AN.

Caring for siblings was an important dilemma encountered during the family admissions, and parents found it often challenging to determine whether siblings should stay at the hospital to maximize treatment participation, or remain at home to maintain regular contact with their school, friends, and activities. Although the unit emphasized awareness of siblings’ needs, parents and patients alike perceived the overall benefit of the treatment for siblings as small to moderate. Adolescent AN greatly affects siblings’ family situation and daily life [49–51], and concern for the welfare of siblings was especially prominent in our study, owing to the severity and chronicity that characterized the patient sample. More research on the perspectives of the siblings of adolescents with AN is important, and in particular, how parents and health services should accommodate the needs of siblings.

## Clinical implications and conclusion

A clinical implication from this study is that family-based inpatient treatment for adolescents with AN may be a useful option when outpatient treatment has proven insufficient. We found that parents, and to some degree former patients, were satisfied with this treatment approach, which supports its feasibility. All families agreed that one or both of the parents would stay at the hospital for the full length of the admission, and dropout was very low (1/58), which also supports the acceptability of the treatment. Although the participation of both parents is crucial in FBT, fathers’ participation level is often low. Our finding that fathers reported a similar, and equally high, level of satisfaction as mothers indicates that the treatment approach was perceived as useful for fathers as well as for mothers, which is likely to promote fathers’ participation.

## Abbreviations

AN: Anorexia nervosa
ED: Eating disorder
EDE-Q: The eating disorder examination questionnaire
FBT: Family-based treatment
GS-PEQ: The Norwegian generic short patient experiences questionnaire
POT: Perception of therapists
TAU: Treatment as usual
